# Novel ZnO hollow-nanocarriers containing *paclitaxel* targeting folate-receptors in a malignant pH-microenvironment for effective monitoring and promoting breast tumor regression

**DOI:** 10.1038/srep11760

**Published:** 2015-07-06

**Authors:** Nagaprasad Puvvada, Shashi Rajput, B.N. Prashanth Kumar, Siddik Sarkar, Suraj Konar, Keith R. Brunt, Raj R. Rao, Abhijit Mazumdar, Swadesh K. Das, Ranadhir Basu, Paul B. Fisher, Mahitosh Mandal, Amita Pathak

**Affiliations:** 1 Department of Chemistry, Indian Institute of Technology, Kharagpur, West Bengal, 721302, India; 2 Department of Pharmacology, Dalhousie Medicine New Brunswick, Dalhousie University, New Brunswick, Canada; 3 School of Medical Science and Technology, Indian Institute of Technology, Kharagpur, West Bengal, 721302, India; 4 Department of Human and Molecular Genetics, Virginia Commonwealth University, School of Medicine; Richmond, VA 23298, USA; 5 Department of Chemical and Life Science Engineering, Virginia Commonwealth University, Richmond, VA 23238, USA; 6 Department of Clinical Cancer Prevention and Systems Biology, University of Texas, MD Anderson Cancer Center, Houston, TX 77030, USA; 7 VCU Institute of Molecular Genetics, Virginia Commonwealth University, School of Medicine, Richmond, VA 23238, USA; 8 VCU Massey Cancer Center, Virginia Commonwealth University, School of Medicine, Richmond, VA 23238, USA; 9 Central Research Facility, Indian Institute of Technology, Kharagpur, West Bengal, 721302, India

## Abstract

Low pH in the tumor micromilieu is a recognized pathological feature of cancer. This attribute of cancerous cells has been targeted herein for the controlled release of chemotherapeutics at the tumour site, while sparing healthy tissues. To this end, pH-sensitive, hollow ZnO-nanocarriers loaded with paclitaxel were synthesized and their efficacy studied in breast cancer *in vitro* and *in vivo*. The nanocarriers were surface functionalized with folate using click-chemistry to improve targeted uptake by the malignant cells that over-express folate-receptors. The nanocarriers released ~75% of the paclitaxel payload within six hours in acidic pH, which was accompanied by switching of fluorescence from blue to green and a 10-fold increase in the fluorescence intensity. The fluorescence-switching phenomenon is due to structural collapse of the nanocarriers in the endolysosome. Energy dispersion X-ray mapping and whole animal fluorescent imaging studies were carried out to show that combined pH and folate-receptor targeting reduces off-target accumulation of the nanocarriers. Further, a dual cell-specific and pH-sensitive nanocarrier greatly improved the efficacy of paclitaxel to regress subcutaneous tumors *in vivo*. These nanocarriers could improve chemotherapy tolerance and increase anti-tumor efficacy, while also providing a novel diagnostic read-out through fluorescent switching that is proportional to drug release in malignant tissues.

Nanomedicine is quickly revolutionizing drug delivery systems (DDS). This is particularly attractive in cancer therapy, as localized delivery to the malignancy is increased, while the negative side effects to a patients’ healthy tissues are circumvented^1^. Various nanocarriers have been proposed for DDS, including inorganic^2^,^3^, composite^4^,^5^,^6^, polymeric vesicles/micelles^7^,^8^,^9^, liposomes^10^ and niosomes^11^,^12^. Many of the proposed nanocarriers rely on passive accumulation, made possible by the unstable vessel architecture of tumors. However, establishing concentrations of chemotherapy in earlier designs of nanocarriers to obtain therapeutic responses have fallen short of achieving this objective due to low drug-capacity and protracted release profiles^13^.

Hollow nanocarriers offer substantial internal space in their core to provide high drug-transport capacity^14^,^15^. Surface modifications of the nanocarrier further enhance the specific targeting to the malignant cells and their microenvironment, regulate release kinetics and improve biocompatibility. Nanocarriers also provide the opportunity to add diagnostic imaging potential to create ‘*theranostics*’. The assessment of nanocarrier bio-distribution often relies on the addition of fluorescent probes. Yet, Genger *et al.* reported photo-bleaching as a major drawback to organic fluorescent probes^16^. The emergence of quantum dots as an alternative resolves photo bleaching and offers unique optical fluorescence properties. Amongst quantum dots, those comprised of ZnO were extensively studied for photophysical, electrochemical and physiological profiles^17^. The intrinsic fluorescence of ZnO, its biocompatibility as a trace element and the ease by which its structure is chemically malleable make it a compelling substrate for nanocarrier design of DDS.

Averting nanoparticle agglomeration, metal oxide toxicity and immune reactivity are essential to obtain good clinical practices (GCP) of DDS fabrications. To mitigate such risks, wherever possible, natural biocompatible polymers should be used for structuring and surface functionalization of the DDS. This was demonstrated recently by Yang *et al.*, using surface tethering of a biocompatible polymer to achieve sustained release of doxorubicin from hollow silica nanoparticles^17^. Similarly, chitosan is an abundant natural biopolymer that can be used as a surface-tethering agent, particularly as new GCP-ready chitosan from non-animal sources become available (ex. MycoDev Inc http://mycodevgroup.com). Chitosan is recognized for its non-toxic, biodegradable, biocompatible and non-immunogenic nature^4^. Ideally, water soluble polymers are used to deliver lipophilic entities/drugs^18^. However, the aqueous insolubility of chitosan has limited its application in nanocarrier fabrication, stimulating new solution-based research. To that end, Pramanik *et al.*, demonstrated that carboxymethylation of chitosan overcomes water insolubility^19^.

In comparison to healthy tissue, the tumor microenvironment is highly acidic. This is due to the nearly 200-fold elevation in glycolysis and lactic acid accumulation from malignant metabolism, cell hyperproliferation and poor perfusion from the torturous vasculature^20^. Sensitizing a nanocarrier to this pH variable is a mechanism to reduce chemotherapy accumulation in healthy tissues and to focus it on the malignant tumors and their microenvironment. We have now designed a chitosan-ZnO nancoarrier that is stable at physiological pH, but collapses to release its drug loaded core in an acidic *milieu*. Click-chemistry renders water-based reactions with no byproducts and is ideal for surface functionalization^21^. Neoplasms, particularly of the breast, express high levels of folate receptors^20^,^22^. For added specificity, our design used click-chemistry to surface conjugate folic acid to facilitate breast cancer cell-specificity synergistically with pH for targeting.

*Paclitaxel*, a member of the taxane drug class, specifically targets microtubule dissociation. It is extensively used for the treatment of human cancers, like breast cancer, malignant lymphoma, osteosarcoma, lymphoblastic leukemia and ovarian cancer^23^,^24^. However, the dose-limiting issues associated with this drug include its poor solubility and toxicity toward normal tissues, which can harm the cardiovascular, nervous, gastrointestinal and hepatic systems. In this context, a controlled and focused release of *paclitaxel* would, in principle, improve the pharmacology and clinical titration of *paclitaxel* thus, enhancing its therapeutic utility.

We have now developed an improved nanocarrier for potential clinical use that addresses several specific issues important to nanocarrier design: process formulation, biocompatibility, specificity, detectability, drug capacity and efficacy. The current design utilizes a combination of pH sensitivity and cell-specific targeting to synergistically improve tumor targeting to overcome off-target effects in healthy tissues with high metabolisms, such as the hepatic and reticuloendothelial systems. Importantly, as an enhanced feature to this design was the increased drug capacity and ability to observe fluorescent photo-switching (blue-to-green) as the nanocarrier is targeted to the tumor. We show that this increases the theranostic value, not only due to the increased fluorescent accumulation of a nanocarrier at the tumor site, but also for determining drug release in proportion to the photo-switching as the nanocarrier breaks down intracellularly. Thus, this allows for future advances to enhance titration as a component of personalized medicine. Lastly, the developed formulation was achieved using biocompatible, water-soluble carboxymethylated chitosan with naturally occurring zinc-oxide and folic acid via click-chemistry techniques. This enhances the potential of the innovation as it improves the processing efficiencies and safety profiles of the constituent materials.

## Materials and Methods

### Materials

Zinc acetate dihydrate (≥98%) and dextrose (99.4%) were purchased from Merck Pvt Ltd, India. 1-chloropropyl amine hydrochloride (98%), chitosan, propargylamine (98%), and *paclitaxel* (PAC, ≥97%) were purchased from Sigma Aldrich St. Louis, MO, USA. N-hydroxysuccinimide (NHS, 98%), N-(3-dimethylaminopropyl)-N-ethylcarbodimide hydrochloride (EDC, 98%), N,N'-Dicyclohexyl Carbodimide (DCC, 99%), folic acid (FA, ≥97%), monochloroacetic acid (≥97%), copper sulphate pentahydrate (CuSO_4_. 5H_2_O, 99.5%), sodium azide (NaN_3_, 99%), sodium ascorbate (98%), and Dimethylsulfoxide (DMSO, 99.2%) were purchased from SRL Pvt Ltd, India. All other chemicals were of analytical grade and were used without further purification unless specified (Additional material and process details can be found as supplemental information).

### Synthesis of carboxymethylated chitosan

Previously, carboxymethylated chitosan (CMC) was prepared using chitosan and a monochloroacetic acid modification^25^. In brief, chitosan and sodium hydroxide (10 g each) were suspended in a 100 ml mixture of isopropanol and water (1:1) followed by heating at 70 °C for 1 h. Monochloroacetic acid (15 g, dissolved in 20 ml of isopropanol) was added drop wise to the resultant mixture for 30 min under vigorous stirring for 4 h at 70 °C. The reaction was terminated by adding 70% ethanol (250 ml) and the product was filtered by washing with 90% ethanol, dried in a vacuum oven for 24 h at room temperature. Finally, the residual product was suspended in acidified ethanol for the formation of carboxymethylated chitosan {^1^H NMR (400 MHz, D_2_O, δ): 1.887 (CH_3_, acetamido group of chitosan), 2.99 (CH, carbon 2 of glucosamine ring), 3.5−4.0 (CH_2_, carbon 3, 4, and 6 of glucosamine ring), 4.18 (CH_2_, carboxymethyl group)}.

### Synthesis of 3-azidopropylamine

The azido derivative of 1-chloropropylamine hydrochloride was prepared as reported previously^26^. Briefly, sodium azide (34.6 mmol) was added to the 3-chloropropylamine hydrochloride solution (11.5 mmol in 20 ml of water) and heated at 80 °C for 15 h. Subsequently, the resultant solution was basified using KOH and extracted with diethyl ether (3 × 25 ml). Additionally, the organic phase was dried over magnesium sulfate and concentrated to result in 3-azidopropylamine as a colorless volatile oil {^1^H NMR (400 MHz, CDCl_3_, δ): 3.35 (t, J = 6.8 Hz, 2H; CH_2_-NH), 2.82 (t, 2H, J = 6.8 Hz, CH_2_N_3_), 1.72 (qn, J = 6.8 Hz, 6.8 Hz, CH_2_), 1.35 (2H, br s, NH_2_)}.

### Synthesis of an alkyne derivative of folic acid

Folic acid (1 mmol) and DCC (1.5 mmol) was dissolved in 10 ml of dry DMSO under inert atmospheric conditions^21^. Activated carbodimide facilely reacts with propargylamine (1.5 mmol) overnight under vigorous stirring in the dark. Lastly, the DCU was removed by filtration and the filtrate was precipitated for the desired alkyne derivative of folic acid by ice cold diethyl ether{^1^H NMR (400 MHz, DMSO-d_6_, δ): 8.62 (s, PtC_7_H, 1H), 8.23–8.29 (d, Pt C6-CH2NH-Ph 1H, J = 20 Hz), 8.02–8.04 (d, CONHCHCO2H, 1H, J = 8 Hz), 7.63–7.71 (d, Ph-C2H and Ph-C6H, 2H), 6.94 (br s, NH_2_, 2 H), 6.6–6.62 (d, Ph-C_3_H and Ph-C_5_H, 2H, J = 8 Hz), 4.45–4.46 (d, PtC6-CH2NH-Ph, 2H, J = 4 Hz), 4.34–4.36 (d, PtC6-CH2NH-Ph), 3.79–3.81 (m, -CONH-CH2CtCH, 2H), 3.06 (t, -CONH-CH_2_CCH, J = 4 Hz), 2.78 (s, CONH-CH_2_CCH, 1H), 2.52 (br s, -OH, 1H), 2.3 (m, -CH_2_CO_2_H), 2.1 (m, -CHCH_2_CH_2_, 1H), 1.7 (m, -CHCH_2_CH_2_, 1H)}.

### Synthesis of Hollow ZnO-nanocarriers

ZnO-nanocarriers were prepared with minor modifications to that previously described^27^. Briefly, 1 mmol of zinc acetate was dissolved in 20 ml of ethanol under vigorous stirring. To this clear solution, 1 g of carbon nanocarriers was suspended and subsequently added with 2 ml of ammonia solution and kept at room temperature for 4 h. By centrifugation, the product was collected and washed twice with water and once with ethanol, then dried at 80 °C for 12 h. The dried product was calcined at 500 °C for 3 h to obtain the hollow ZnO-nanocarriers (HZnO).

### Synthesis of carboxyl functional group decorated on ZnO-nanocarriers

Initially, we dissolved 20 mg of CMC in 5 ml of de-ionized water. To this, 100 mg of HZnO-nanocarriers were added to obtain a homogeneous mixture, which became opalescent upon the addition of ethanol, indicating the formation of carboxyl functional groups on the HZnO surface.

### Synthesis of Azide group functionalized ZnO-nanocarriers

Briefly, HZnO (1 mmol of carboxyl groups concentration as measured by NaOH titration) and 1.5 mmol of EDC and NHS were dissolved in 10 ml of dry DMSO under inert atmosphere to activate carbodimide and 1.5 mmol of 3-azidopropargylamine was then added and kept overnight under vigorous stirring. The desired azide derivative of chitosan was collected by cold ethanol centrifugation, AZnO.

### Conjugation of folic acid on ZnO surface through click chemistry

The alkyne derivative of folic acid (1 mmol) and 3-azidofunctionalized ZnO-nanocarriers (100 mg) were suspended in a mixture of water and DMSO (1:1). To this solution 110 μl of sodium ascorbate (1 M) as well as 175 μl of copper sulphate pentahydrate (1 M) was added simultaneously and stirred overnight in the dark. The product was separated by centrifugation and washed with ethanol, to finalize the nanocarrier, FCZnO. A similar procedure was repeated with *paclitaxel* (PAC) loading during synthesis to create the final DDS, designated as FCPZnO.

### Hemocompatibility study

In this study, free PAC, HZnO and FCPZnO-nanocarriers, were individually suspended in phosphate buffer saline (PBS). Hemocompatibility of these samples were analyzed as described previously^3^. Data is reported as mean ± SD (n = 3).

### Cellular uptake studies by epifluorescence microscopy and flow cytometry

Due to the inherent luminescent properties of the nanocarrier they served as an intrinsic fluorescence probe to efficiently explore uptake of FCPZnO. Briefly, MCF-7 cells were seeded on glass cover slides in 60-mm plates at a density of 1 × 10^4^ cells per coverslip and were allowed to adhere. These cells were then treated with FCPZnO for 1, 2, 3, 6 and 12 h at 37 °C, 5% CO_2_ incubation. Cells were rinsed with PBS three times, then fixed with 70% ethanol for 20 min at 37 °C and visualized with an epifluorescence microscope (Leica Microsystems GmbH, Germany) equipped with an argon laser using FITC filter (λ_Ex_ 488 nm and λ_Em_ 525 nm).

To further validate the intracellular uptake of FCPZnO we assessed fluorescence by flow cytometry. MCF-7 and MDA-MB-231 cells seeded at a density of 5 × 10^4^ cells per 60-mm plate were allowed to grow to 70% confluence, then treated with FCPZnO at varying time intervals (3, 6 and 12 h). At the end of the incubation period, the cells were collected and washed three times with PBS to wash excess FCPZnO. Nanocarrier uptake was analyzed in the green FL1 channel (λ_Ex_ 490 nm, λ_Em_ 520 nm) and data were collected from >10,000 gated events, then analyzed using the CELL Questpro software program (BD Biosciences, USA).

### Changes in cell surface potential

The nanocarrier-cell interaction was revealed by determining the surface charge of nanocarrier-treated MCF-7 cells using zeta-potential analysis at 25 °C as described previously^28^. Cells were grown and incubated with 100 μg/ml FCPZnO at varying time intervals from 0 to 3 h with surface charge monitoring of the FCPZnO with cells.

### Cell viability assay

MTT-assay for MCF-7 and MBA-MB-231 cytotoxicity was used, as before^29^,^30^. Briefly, 100 μl of 2 × 10^3^ cells were seeded into each well of a 96-well plate. Corresponding PAC, HZnO and FCPZnO ranging 0.001–40 nM were added to each well. After 48 h incubation at 37 °C the nanocarriers were washed and replaced in 100 μL of media containing MTT (1 mg/ml); absorbance was measured at 570 nm.

### Cell cycle and apoptosis analysis

Flow cytometry analysis of DNA content was performed to assess the cell cycle phase distribution, as described previously^31^. In brief, both attached and floating cells were harvested by trypsinization after a 48 h treatment with the respective IC_50_ value 14.02 ± 0.6680 to 8.060 ± 0.4788 nM, respectively, in MCF-7 cells and 11.84 ± 0.5803 to 7.213 ± 0.2847 nM, respectively, in MDA-MB-231 cells of native PAC and FCPZnO; then washed twice with PBS (pH 7.2) and incubated in 70% ethanol, kept at −20 °C for overnight fixation. Subsequently, cells were stained for DNA content using propidium iodide (PI) and analyzed using CELL Questpro software (BD Biosciences, USA) to determine the cell cycle phase distribution and apoptosis.

### Morphological analysis

MCF-7 cells were seeded at a density of 6 × 10^3^ on glass coverslips and treated without (control), or with PAC, HZnO and FCPZnO for 48 h. Post-incubation, the cells were washed three times in 0.1 M cacodylate buffer (pH 7.4) and then fixed in ice-cold 1% OsO_4_ for 1 h with additional EM-processing as described previously^4^.

For nuclear analysis, cells were grown as above and fixed with formaldehyde, then imaged by fluorescence microscopy(Leica DMR, Germany) with DAPI as before^32^.

### *In vivo* Xenograft Studies

Tumor responses to PAC, HZnO and FCPZnO samples were studied using a human breast cancer nude mouse xenograft model. All procedures in animal studies were approved and performed in accordance with the institutional animal use and investigation committee at Virginia Commonwealth University, School of Medicine, Richmond, VA, USA. Mice were housed and acclimatized in a pathogen free environment at the institute animal facility for 1 week prior to injection with MDA-MB-231 cells. Exponentially growing MDA-MB-231 cells were collected and 2.5 × 10^6^ cells in Matrigel (0.5 mg/mL) were injected subcutaneously (s.c.) in 6–7 week-old female athymic BALB/c (nu+/nu+) mice^4^,^33^,^34^. After 6 days, all of the mice were then weighed, the tumors measured using microcalipers to calculate tumor volume^35^. All mice were randomized into four groups, containing 3 mice per group. The groups included vehicle (PBS), empty HZnO-nanocarriers, and drug-loaded nanocarriers FCPZnO at an equalized dose to the drug-alone *paclitaxel* (PAC) group {10 mg/kg/day of body weight, i.v. twice weekly for 5-weeks}. Weight was monitored to ensure mice did not become cachexic and tumors were measured weekly with calipers.

### *In vivo* fluorescence imaging

To ensure traceability in deep tissue, nanocarriers were tagged with an IR-Dye 680 to form IR-680-FCPZnO. Mice bearing human MDA-MB-231 tumor xenografts were injected either with IR-680 dye or IR-680-FCPZnO. Whole-body optical imaging was performed using IVIS Spectrum (Pre-clinical *In vivo* Imaging System, Perkin Elmer, MA, USA) in fluorescence mode. Mice were subjected to anesthesia using inhalant isoflurane (Henry Schein Animal Health, Dublin, OH, USA) prior to and during the imaging procedure. During the period of image acquisition, the mice were positioned within the scanner of the imaging platform with IVIS Flow switched on, that regulated the flow of oxygen, isoflurane and removed the waste isoflurane gases by anesthesia gas filter unit (f/air) (A.M. Bickford Inc., NY, USA).

### Immunohistochemical analysis

Analysis of PAC, HZnO and FCPZnO samples were qualitatively assessed for proliferation, angiogenesis and apoptosis of human breast cancer xenografted mice tumors using Ki67 and CD31 antibodies and TUNEL nuclei staining. Tissue specimens were processed for immunohistochemical analyses as described previously^4^,^36^.

### Statistical analysis

Statistical analysis was performed by GraphPad Prism 5 software. Data were presented using mean ±SD. The statistical significance was determined by using one-way analysis of variance (ANOVA). ***P < 0.001 and **P < 0.05 were considered significant.

### Results & Discussion

**Figure 1:**
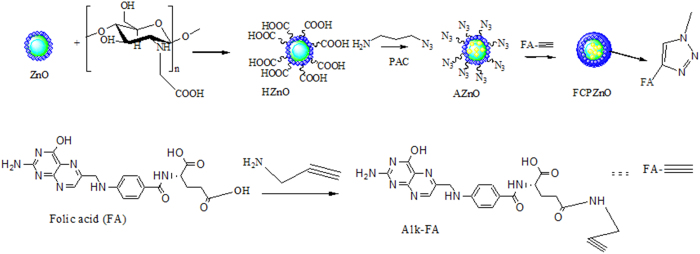
Schematic representation of the approach used to functionalize folic acid through the click chemistry approach for the *paclitaxel* (PAC) encapsulated ZnO spheres.

A schematic illustration of ZnO-nanocarrier chemistry is shown in Fig. 1.The protruded azide groups on ZnO surface and folic acid conjugate through click chemistry on PAC loaded ZnO-nanocarriers highlights our material process of making DDS. Initially, carbon spheres were suspended in zinc acetate solution, and with the addition of ammonia; resulted in the formation of zinc hydroxide on the surface of carbon spheres. Embedding of the hollow natured ZnO-nanocarrier was achieved through pyrolysis of carbon.

**Figure 2:**
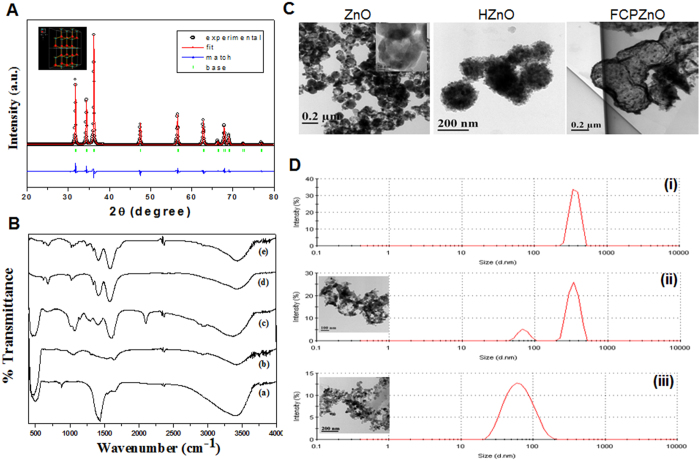
Characterization of nanocarriers (**A**) XRD pattern of ZnO, (**B**) FTIR spectrum of ZnO (i), HZnO, AZnO (iii), FCZnO (iv), FCPZnO (v). (**C**) Transmission electron micrographs of ZnO, HZnO and FCPZnO. (**D**) Hydrodynamic radius of FCPZnO samples at pH 7 (i), and 5.2 pH incubation time interval 3 h and 6 h (iii) Results are representative of three independent experiments.

The phase analysis of ZnO was confirmed by powdered XRD (Fig. 2A) with depicted peaks at 31.7, 34.5, 36.2, 47.49, 56.58, 62.7, 66.3, 67.9, 69.1, 72.53 and 76.9, and respective reflection crystal planes were indexed to be (100), (002), (101), (102), (110), (103), (200), (112), (201), (004) and (202). From the above data, we confirmed the hexagonal structure of the sample with P63mc symmetry, which was consistent with standard JCPDS file no. 36–1451^37^ and a corresponding crystal structure obtained from Rietveld analysis (Fig. 2A inset). The hollow nanocarriers have higher entrapment capacity for greater concentrated release of drugs^38^.

To evaluate the functional groups of the nanocarrier surface following conjugation of folic acid, FTIR analysis was performed (Fig. 2B). ZnO-nanocarriers exhibited an intense broad band at the 430–500 cm^-1^ region due to the presence of a Zn-O stretching mode. The HZnO depicted clear bands for symmetric and asymmetric stretching vibrations of the free carboxylic groups at 1406 and 1633 cm^-1^, respectively. Further, the C–O stretching vibrations resulted in bands at 1023 and 1069 cm^-1^, while bands at 673 and 3429 cm^-1^ represent O-H stretching vibrations and a bridged oxygen (C-O-C) was observed at 1160 cm^-1^. Surface treatment of HZnO by 3-azidopropyl amine through EDC coupling and corresponding functional groups are illustrated in Fig. 2B. However, AZnO showed an asymmetric stretching of N = N = N at 2065 cm^-1^ and amide linkage at 1646 and 1550 cm^-1^ of amide I and amide II, respectively^21^.

The successful immobilization of folic acid on AZnO through click chemistry indicates the disappearance of a peak at 2111 cm^-1^ and the observed characteristic peaks of folic acid at 1603 and 1656 cm^-1^ in the FCZnO sample. Further, FCPZnO generated a distinctive peak at 1736 cm^-1^ indicating the presence of the ester group of PAC. In addition, we also confirmed the successful conjugation of folic acid via click chemistry conjugation by ^1^H NMR and identified peaks in the range of 2.8 to 3.5 ppm ((i)^15^, (iii)), which is due to the presence of glucosamine moieties. An additional peak around 8.12 ppm (iv) is due to triazole formation of the click reaction (Fig. S1)^26^. In addition, we identified aromatic protons of folic acid peaks at 8.37 (v), 7.80 (vi), 7.57 (vii), 6.38 (viii), and 6.21 (ix) ppm indicating that a successful conjugation of folic acid through click chemistry was accomplished.

TEM imaging revealed that ZnO-nanocarriers produced through basic hydrolysis in the presence of carbon spheres under pyrolysis formed an assemblage of ZnO particles encircled over carbon spheres (Fig. 2C). The mean size of hollow ZnO-nanocarriers was ~125 nm with a shell thickness of~16 nm authenticated by TEM analysis. The architecture of ZnO-nanocarriers was retained following sonication indicating its stability, as visualized by TEM micrographs (Fig. 2C ZnO inset). Previously, a similar morphology was observed by Wang *et al.* from silica-colloidalsomes^39^.

Tethering and drug encapsulation of nanocarriers resulted in incremental size increases of HZnO (180–220 nm) and FCPZnO (200–310 nm) samples. From DLS measurements, FCPZnO showed its intense peak at 362 nm (Fig. 2D (i)) under neutral pH (7.2). However, we observed two consecutive peaks at 342 and 75 nm (Fig. 2D (ii)) after a 3 h incubation at around pH 5.2 (endo-lysosomal pH). The peak at 342 nm signifies the antecedent-state of disorganized nanocarriers at the initial phase, while the peak at 75 nm revealed disorganized ZnO particles. Following, prolonged incubation (6 h), the intensity of the 75 nm peak was amplified, while concomitantly the 342 nm peak disappeared, thus signifying the complete collapse of the hollow nanocarriers (Fig. 2D (iii)). Analogous results were observed in the case of HZnO-nanocarriers (data not shown). These results established that these nanocarriers are pH-sensitive. Further, these results were supported by TEM studies (Inset of Fig. 2D (ii and iii)). The collapse of the hollow natured nanocarriers in an acidic environment provides an additional benefit for payload delivery in the acidic environment of malignant cells.

**Figure 3:**
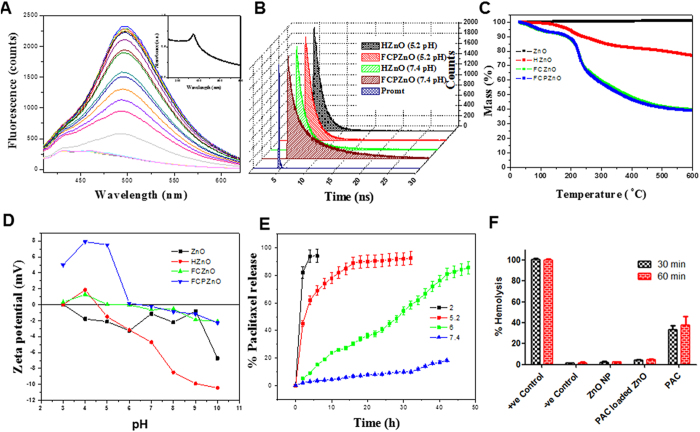
(**A**) Photoluminescence spectra of FCPZnO at a pH 5.2 at various time periods up to 6 h and corresponding absorption spectra as an inset, (**B**) time correlated single photon counting data of emission wavelength 495 nm and excitation wavelength 375 nm for HZnO and FCPZnO samples at various pHs of 5.2 and 7.4, (**C**) thermo gravimetric analysis of ZnO, HZnO, FCZnO and FCPZnO samples and their corresponding zeta potential in (**D**), (**E**) drug release studies, (**F**) Hemolytic assay of PAC, HZnO and FCPZnO. –ve control of 0% lysis (in 1x PBS) and + ve control of 100% lysis (in 1% Triton X-100) were employed in this experiment. All the samples (excluding PAC) showing insignificant amount of hemolysis with respect to the +ve control. The bars indicate the means ± SD (n = 3).

Fluorescence is a constitutive physical property of ZnO (Fig. 3A). Absorbance and fluorescence of FCPZnO-nanocarriers were investigated by UV-Vis and fluorescence spectrophotometric studies. In agreement with previous literature, the absorption maximum of FCPZnO was 374 nm (Fig. 3A inset)^27^. Moreover, the fluorescence spectra of nanocarriers exhibited blue and green emission maxima at 434 and 495 nm, respectively. At neutral pH, an emission maximum of 434 nm intensity was higher than that of 495 nm. Under acidic conditions, we observed the disappearance of the 434 peak with simultaneous proportionate increase of the 495 peak maxima Fig. 3A. This demonstrated the fluorescence-switching feature of these nanocarriers. At this pH, augmentation of fluorescence (495 nm) was 10-fold more compared to neutral condition (Fig. 3A). Furthermore, increased fluorescence intensity correlated with a decrease in particle size from 230 nm to 35 nm indicating the disruption of the nanospherical architecture^40^,^41^,^42^. Thus, the fluorescence intensity is inversely proportional to particle size. Further, fluorescence decay studies of FCPZnO revealed two life-time components at 1.27 and 5.42 ns for 495 nm under physiological conditions (Fig. 3B). In acidic pH (5.2), the amplitude and lifetime of the shorter component decreases, while the longer component increases. Therefore, the peak maxima at 494 and 434 nm correspond to longer and shorter lifetime components respectively. Similar results were also observed in the case of HZnO-nanocarriers.

Thermogravimetric spectrum analysis revealed no disparity in the weight of naked ZnO-nanocarriers even in absence of carbon spheres (Fig. 3C). HZnO showed weight loss of 28% at 600 °C due to the presence of surface tethered CMC, while FCZnO (i.e., absence of PAC in ZnO-nanocarriers conjugated with FA) and FCPZnO (i.e., PAC loaded ZnO-nanocarriers conjugated with FA) displayed reductions in weights of 60 and 62%, respectively; the differential weight reduction between the two accounts for the successful entrapment of the drug (Fig. 3C). The surface charge of naked nanocarriers was determined by zeta potential studies in a wide range of pH conditions (3 to 10). Naked nanocarriers exhibited a positive charge of 0.0348 mV at pH 3, any further increase in pH produced a net negative charge with the isoelectric point of ZnO at 3.1 (Fig. 3D), whereas HZnO showed an isoelectric point at 4.5. However, the surface of FCZnO exhibited a positive charge below the isoelectric point (pH 5.4) but above 5.4 the surface charge became negative. Similar results were also observed in the case of FCPZnO, with an isoelectric point 6.4, as the presence of excess positive charge was gained due to drug entrapment. The positivity of nanocarriers in acidic and neutral environments favor its use in drug delivery applications for cancer^43^ therapy. The encapsulation efficacy of the FCPZnO was determined to be approximately 82%.

Collectively, based on the above results, we hypothesized that healthy negatively charged cells at physiological pH would electrostatically repel HZnO-nanocarriers, thus reducing the interaction with normal healthy tissues. This could result in a reduction of drug-induced side effects. In the circulation, negatively charged nanocarriers become positively charged when encountering an acidic tumor microenvironment, thus, manifesting an electrostatic attraction to malignant cells and accumulation of nanocarriers^44^.

### Drug release profile

Regulated drug-release minimizes side effects and avoids toxicity in normal cells. Here, the disorganization of nanocarriers favors a pH-regulated release of drug to meet the therapeutic index at the tumor site. The drug release profile from FCPZnO at physiological pH was 18% after 32 h, but at pH 5.2 the release percentage was 74% after just 6 h incubation (Fig. 3E). At an intermediate pH of 6, 51% release was achieved by 45 h of incubation. These results were in agreement with our TEM and DLS analysis. Additionally, these results are in agreement with the fluorescent-switching intensity measures (Fig. S2).

### Hemocompatibility study

Assessment for hemolysis is a prerequisite for intravenous administration to patients. Structural constituents, drugs or physical associations with RBC can negatively impact hemostability^4^,^45^. Here, PAC alone caused significant hemolysis, likely due to drug toxicity (Fig. 3F). However, an equivalent concentration encapsulated PAC as FCPZnO, or the empty nanocarrier HZnO, demonstrated very low hemolysis (<2%), well below the toxicity threshold of <5%, suggesting both integrity of the construct and repulsive forces between the nanocarrier and the RBC membrane at a neutral blood pH^46^.

### Assessment of cellular uptake by epifluorescence microscopy and flow cytometry

**Figure 4:**
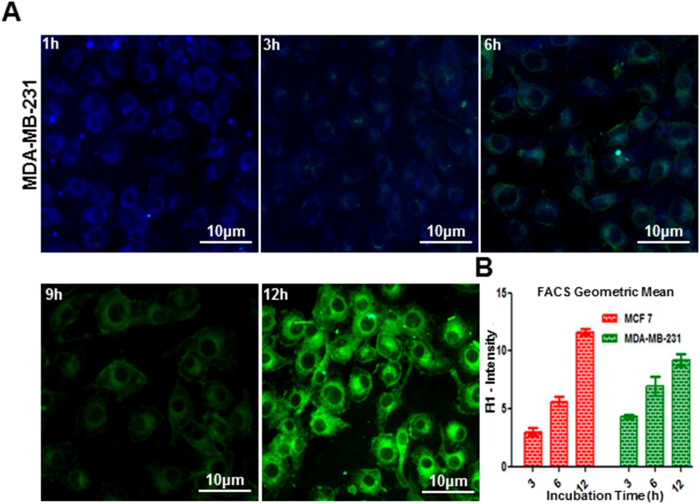
Cellular uptake studies by flow cytometric and epi-fluorescence microscopic studies. Cells treated with FCPZnO (100 μg/ml) and incubated at varying time intervals. (**A**) Qualitative analysis of cellular localization of nanoparticles in MDA-MB-231 by epi-fluorescence microscopy for 1, 2, 3, 6 and 12 h (scale bars: 10 μm at 20x). (**B**) Geometric mean analysis of nanocarriers cellular uptake at 3, 6 and 12 h through flowcytometry in MCF-7 and MDA-MB-231. Results are representative of three independent experiments.

A time-dependent uptake study was carried out using FCPZnO to assess nanocarrier accumulation inside tumor cells. The abundant folate receptors present on breast cancer cells augment receptor-mediated endocytosis when nanocarriers are surface functionalized with folic acid. Using the intrinsic fluorescence property of these nanomaterials, analysis of cellular uptake behavior was performed in MCF-7 and MDA-MB-231cells by fluorescence microscopy and flow cytometry. Following 1 h incubation, cells appeared blue in colour resulting from initial indistinct nanosomal organisation following uptake (Fig. 4A). However, cells gradually shifted to bluish green due to endolysosomal nanocarrier disorganisation by the 3 h time point, and then sequentially further shifted into the green spectrum by 6, 12, and 24 h. This suggests that uptake of nanocarriers would result in drug release intracellularly and would follow the blue-green shift as nanocarriers disorganize.

In flow cytometric analysis, the mean fluorescence intensity (MFI) of MCF-7 and MDA-MB-231 cells increased progressively with incubation time, peaking at 12 h, which was further supported by the summation of multiple geometric mean analyses (Fig. 4B).These observations are in agreement with our fluorescence spectrophotometric and decay studies above. Further, zeta-potential studies of MCF-7 cells reveal altered surface potentials from control (−6.3 mV) when incubated with FCPZnO for 30 mins (−6.11 mV), 1 h (−2.53 mV), 2 h (−2.42 mV) and 3 h (−2.36 mV). This change in surface potential with respect to control, confirms the uptake of nanocarriers by cellular endocytosis. The cellular uptake induced fluorescence shifting from blue to green is expected based on the cell free assessments and the expected pH shift in the acidic endo-lysosomal compartments^47^.

### Cytotoxic effects of PAC and FCPZnO on breast cancer cells

**Figure 5:**
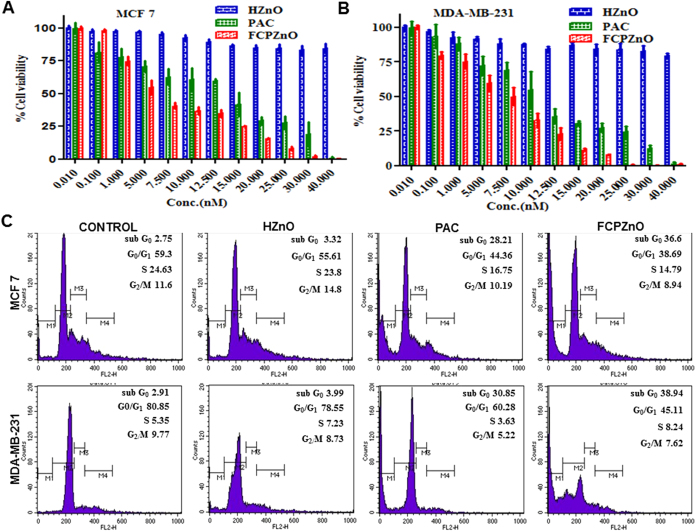
Proliferation assays after PAC, HZnO and FCPZnO treatment in (**A**) MCF-7, (**B**) MDA-MB-231 cells was measured using MTT assays. (**C**) Apoptotic activity of control, PAC, HZnO and FCPZnO-nanocarriers on MCF-7 and MDA-MB-231 cells by phase distribution study. Results are representative of three independent experiments.

To evaluate the *in vitro* anti-proliferative effect of PAC, HZnO and FCPZnO-nanocarriers, MTT dye reduction assays were performed and mitochondrial function was assessed. After a 48 h incubation of cells with PAC, HZnO and FCPZnO, significant growth suppression was observed with drug (r = −0.461, P < 0.05). PAC at 35 nM concentration exhibited < 25% growth inhibition, while at an equivalent concentration, FCPZnO significantly decreased cell proliferation to <5%, likely as a result of intracellular endo-lysosomal release of PAC (Fig. 5A,B). The IC_50_ values of PAC and FCPZnO were found to decrease from 14.02 ± 0.6680 to 8.060 ± 0.4788 nM, respectively in MCF-7 cells and 11.84 ± 0.5803 to 7.213 ± 0.2847 nM, respectively in MDA-MB-231 cells. The FCPZnO demonstrated a significantly higher proliferation inhibitory effect as compared to PAC. This is likely a result of both an enhanced uptake due to folate receptor endocytosis and intracellular release within the acidic endo-lysosome.

### DNA content analysis

The effect of PAC, FCPZnO and drug-free nanocarriers on MCF-7 and MDA-MB-231 DNA content distribution was analyzed for cell cycle status and sub-G apoptosis (Fig. 5C). Both PAC and FCPZnO demonstrated reduced proliferative states and large increase in sub-G apoptosis. The anti-proliferative efficacy of FCPZnO was greater in MCF-7 than MDA-MB-231. However, there was approximately 10% more total apoptosis in FCPZnO treated in both MCF-7 and MDA-MB-231cells, as compared to PAC alone. HZnO-nanocarriers exhibited negligible effects on the cells. These results indicate that FCPZnO, probably due to internalization by folate receptor mediated endocytosis, is more efficacious than PAC alone *in vitro*.

### Morphological analysis

**Figure 6:**
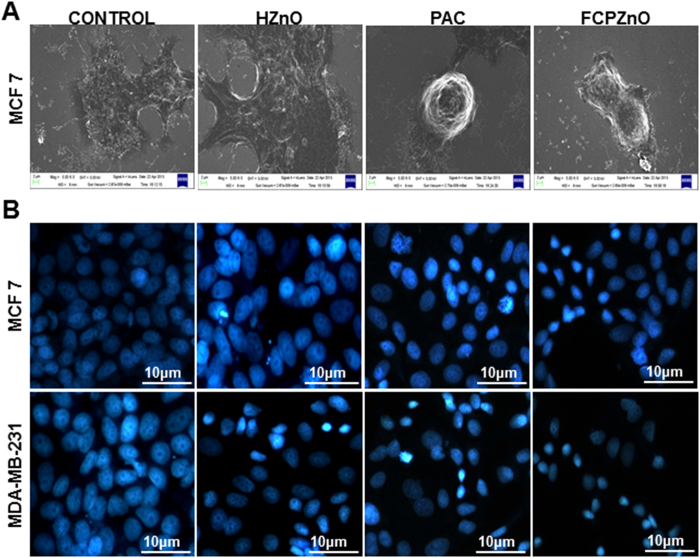
(**A**) Scanning electron microscopic images of MCF-7 cells treated with control, HZnO (equivalent to weight of the PAC nanoformulation), PAC (IC_50_) and FCPZnO (IC_50_) for 48 h. In control cells, healthy filopodia and lamellipodia of MCF-7 cells are observed while truncated cytoplasmic extensions (lamellipodia and filopodia) in PAC and FCPZnO are visualized. (**B**) Nuclear morphological analysis of Control, HZnO, PAC and FCPZnO treated MCF-7 and MDA-MB-231 cells by DAPI staining (epi-fluorescence microscopic image at 20x and scale bars: 10 μm). Results are representative of three independent experiments.

To assess the cytotoxic effects of PAC and FCPZnO in MCF-7 and MDA-MB-231 cells, SEM and DAPI staining were performed. Cell morphology by SEM demonstrated complete loss of cellular cytoskeleton assemblies as revealed by the absence of cellular protrusions and thickening of the cell body in PAC treated cells, and this was observed more often in FCPZnO treated MCF-7 cells (Fig. 6A).

Control and HZnO-nanocarriers showed only rarely condensed nuclei, typical of cells *in vitro*, where a majority of cells show large, flattened nuclei with clear nucleoli (Fig. 6B). Early indications of apoptosis were observed in PAC and to a greater degree in FCPZnO by DAPI analysis of nuclear morphology. Nuclei were mostly condensed, indicative of DNA fragmentation. Further, virtually no nucleoli were observed in FCPZnO nuclei.

### *In vivo* fluorescence imaging

**Figure 7:**
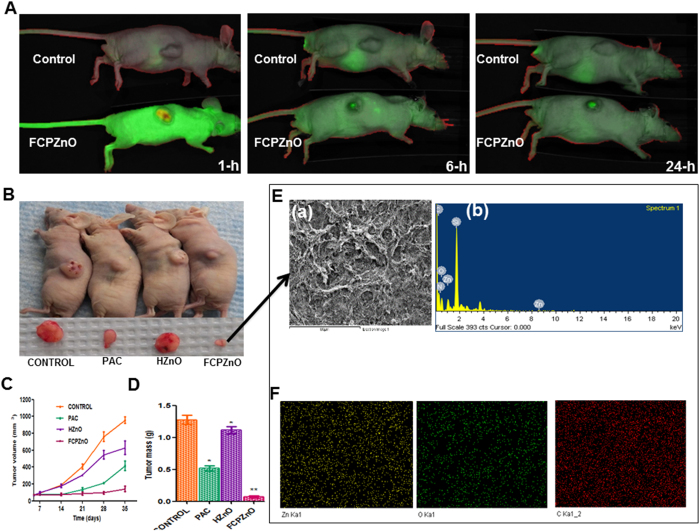
Regression of MDA-MB-231 tumor xenografts in mice treated with FCPZnO and PAC. (**A**) *In vivo* real-time images of IRDye 680-labeled FCPZnO compared to control (IRdye-680). (**B**) Drug treatment groups received PAC (10 mg/kg, i.v.), equivalent dose of PAC in nanoformulation (10 mg/kg, i.v.), and equivalent weight of nanocarrier (i.v.). Tumor volumes in the FCPZnO group were significantly diminished in comparison with free PAC at 35 days after treatment (P < 0.05). (**C**) Tumor volume (**D**) Tumor mass (**E**) spectral shift of tumor sections of FCPZnO-treated mice (a and b). (**F**) FESEM image of tumor cross section area for EDX elemental mapping of corresponding sample zinc (yellow), oxygen (green) and carbon (red) and corresponding EDX spectra.

To ascertain whether the nanocarrier accumulates efficiently in tumors, we tagged FCPZnO with IR-680 nm dye prior to intravenous delivery in BALB/c (nu+/nu+) mice with human MDA-MB-231 breast cancer xenografts. IR680-FCPZnO (5 mg/kg body wt.) was injected systemically by tail vein injection, and the mice were imaged over time using an *in vivo* imaging system (IVIS Spectrum) in fluorescence mode. *In vivo* Fluorescence imaging (FLI) was done using a spectral unmixing approach to eliminate or reduce tissue auto-fluorescence. The composite figure shows the net fluorescence due to IR680 dye bound with FCPZnO with subtracted auto-fluorescence from the tissue (Fig. 7A). Sequential observations at different time points revealed an initial systemic bio-distribution, but with intense signals developing over time that were clearly associated with the subcutaneous tumors (Fig. 7A). These results substantiate our *in vitro* cellular data and support a targeted delivery of the FCPZnO in established tumors *in vivo*. Thus, we conclude that the nanocarriers accumulated at the tumor site due to a combination of preference for folate surface functionalization of the breast cancer cells and the charge repulsion of the nanocarriers to healthy tissues.

### Effective role of FCPZnO in reducing MDA-MB-231 xenograft tumors in nude mice

**Figure 8:**
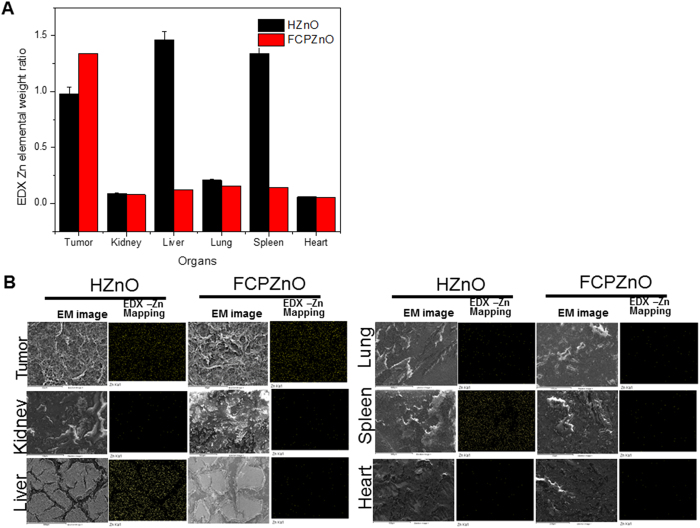
(**A**) Tissue sections of organs from mice treated with HZnO and FCPZnO were subjected to EDX mapping analysis for assessing retention ability of Zn nanocarrier. Compared Zn EDX elemental mapping with HZnO and FCPZnO samples treated with mice of various organs like tumor, kidney, liver, lung, spleen and heart (**B**). The changes were even more prominent when animals were treated with FCPZnO nanocarriers. Results are representative of three independent experiments.

*In vitro* results suggested equal or better anti-proliferative and cytotoxic effects using therapeutic doses of *paclitaxel* delivered by FCPZnO. Based on the *in vivo* tracing studies, FCPZnO is indicated to accumulate intra-tumorly within hours of delivery (Fig. 7A). Follow-up studies of tumor volume and mass however, clearly demonstrated more than 3-fold greater efficacy in tumor regression favouring the FCPZnO delivery compared to an equal dose of PAC in human MDA-MB-231 breast cancer xenografts (Fig. 7B, C and D). A slight but significant reduction in tumor mass was observed in HZnO treated animals; whether this is due to established low zinc bio-availability in malignant tissue remains to be determined. To confirm the accumulated presence of FCPZnO, tumors were analysed by detailed spectral mapping using energy-dispersive X-ray (EDX) spectroscopy (Fig. 7E(a and b)). EDX mapping revealed the presence of zinc (yellow), oxygen (green) and carbon (red) elements within the tumor and its corresponding morphology (Fig. 7F) was determined through FESEM for corresponding spectra. To establish whether the combination of pH-sensitive uptake with folate-receptor targeting increased the nanocarrier accumulation in the tumor site we compared Zn accumulation between equal sections of HZnO and ZCPZnO animals (Fig. 8 (A,B)). Further, to determine whether the additional folate targeting prevented any aberrant off-targeting of other organs we also examined non-tumor tissues. The addition of folic acid targeting, nanocarrier accumulation in the tumor site increases by ~30%, whereas it reduces off-target accumulation in the liver and spleen by >80%. The combined results establish a strong *in vivo* connection for a favoured accumulation of FCPZnO regulated drug release by nanocarrier disruption in the acidic tumor microenvironment for greater tumor regression and reduced off-target accumulation due to pH sensing alone.

Further, we assessed the effects of FCPZnO-nanocarriers on proliferation, and angiogenesis by Ki-67 (a marker of cellular proliferation), and CD31 (a marker of angiogenesis), respectively on mice xenograft tumors. PAC and FCPZnO-nanocarrier-treated tumors showed reduced expression of Ki 67 and CD31 in comparison to control (Supplemental Information), which is consistent with previous reports using the native drug^48^. Control tumors prominently showed hypercellular areas with elevated Ki67 and CD31expression, while HZnO treated tumors were minimally affected. This is in contrast to FCPZnO treatments, demonstrating reduced cellular proliferation and angiogenesis. This can be attributed to the effect of nanocarrier delivered PAC.

## Conclusions

Here we establish a ‘*theranostic*’ nanocarrier engineered with biocompatible substrates that show preferential bioaccumulation and cancer-cell uptake by surface functionalization with folic acid, which are capable of regulated unloading of chemotherapy in the acidic, malignant microenvironment. This report is the first to document disruption of hollow ZnO-nanocarriers that show parallel drug release with a fluorescence reporting mechanism. The potential clinical translatability of our fluorescent photo-switching is a unique advantage in the design or our nanocarrier, as it could provide a diagnostic-based titration of chemotherapy and opportunities for future patient-tailored therapy in personalized medicine. The use of click-chemistry is an ideal mechanism to advance cancer-specific surface functionalization, so as to provide broader off-the-shelf potential for the design of novel nanotherapeutics more specifically, for different cancer types. Using a carbon-based template, calcinations and a soluble chitosan derivative ensures stability and biocompatibility, with significantly greater drug-load capacity to achieve strong anti-tumor effects using a practical and achievable dosing regimen; all with reduced side-effects by preventing drug accumulation in other organs. In particular for chemotherapeutic agents, such as *paclitaxel* that display negative off-target side effects, a controlled *in vivo* release that provides higher tumor site concentrations holds significant potential to enhance the therapeutic efficacy of chemotherapy. Achieving greater therapeutic control to regress tumors by using site-directed means of release, significantly de-risks drugs and doses that would otherwise compromise healthy tissues. Overall, we are encouraged by this ‘smart-targeting of chemotherapy’ for its overall promise to improve the quality of life, recovery and outcome of patients with cancers of the breast, and potentially other organs.

## Additional Information

**How to cite this article**: Puvvada, N. *et al.* Novel ZnO hollow-nanocarriers containing *paclitaxel* targeting folate-receptors in a malignant pH-microenvironment for effective monitoring and promoting breast tumor regression. *Sci. Rep.* **5**, 11760; doi: 10.1038/srep11760 (2015).

## Supplementary Material

Supplementary Information
