# Supplementary material for: Novel ZnO hollow-nanocarriers containing paclitaxel targeting folate-receptors in a malignant pH-microenvironment for effective monitoring and promoting breast tumor regression
Source: Sci Rep. 2015 Jul 6;5:11760. doi: 10.1038/srep11760 (PMC4491843; doi:10.1038/srep11760)
Supplement: Supplementary Information [file srep11760-s1.docx]

**Novel ZnO hollow-nanocarriers containing *paclitaxel* targeting folate-receptors in a malignant pH-microenvironment for effective monitoring and promoting breast tumor regression**

Nagaprasad Puvvada, ^1,2^ Shashi Rajput,^3^ B.N. Prashanth Kumar,^3^ Siddik Sarkar,^4^Suraj Konar,^1^ Keith R. Brunt,^2^ Raj R. Rao,^5^ Abhijit Mazumdar,^6^ Swadesh K. Das, ^4,7,8^ Ranadhir Basu,^9^ Paul B. Fisher, ^4,7,8^ Mahitosh Mandal,^3^* and Amita Pathak,^1^*

^1^Department of Chemistry, Indian Institute of Technology, Kharagpur, West Bengal, India, 721302

^2^Department of Pharmacology, Dalhousie Medicine New Brunswick, Dalhousie University, New Brunswick, Canada.

^3^School of Medical Science and Technology, Indian Institute of Technology, Kharagpur, West Bengal, India, 721302

^4^Department of Human and Molecular Genetics, Virginia Commonwealth University, School of Medicine; Richmond, VA23298, USA.

^5^Department of Chemical and Life Science Engineering, Virginia Commonwealth University, Richmond, VA, 23238, USA

^6^Department of Clinical Cancer Prevention and Systems Biology, University of Texas, MD Anderson Cancer Center, Houston, TX 77030, USA

^7^VCU Institute of Molecular Genetics, Virginia Commonwealth University, School of Medicine, Richmond, VA, 23238, USA

^8^VCU Massey Cancer Center, Virginia Commonwealth University, School of Medicine, Richmond, VA, 23238, USA

^9^Central Research Facility, Indian Institute of Technology, Kharagpur, West Bengal, India, 721302

*Correspondence to: Prof. Amita Pathak, Department of Chemistry, Indian Institute of Technology, Kharagpur, West Bengal, PIN-721302, INDIA. Fax: +91-3222-255303, +91-3222 – 283312. E-mail: ami@chem.iitkgp.ernet.in Email: [ng266711@dal.ca](mailto:ng266711@dal.ca)

*Correspondence to: Prof. Mahitosh Mandal, Schoolof Medical Science and Technology, Indian Institute of Technology, Kharagpur, West Bengal, PIN-721302, INDIA. Fax: +91-3222-282221.Tel: +91-3222-283578, E-mail: [mahitosh@smst.iitkgp.ernet.in](mailto:mahitosh@smst.iitkgp.ernet.in)

*Abbreviations:*

DDS = drug delivery systems

ZnO = zinc-oxide

GCP = good clinical practices

PAC = *paclitaxel*

CMC = carboxymethylated chitosan

AZnO = Azide derivative chitosan hollow Zn-Oxide nanocarriers

HZnO = Hollow Zinc-Oxide nanocarriers

FCZnO = Folic-acid Conjugated hollow Zinc-Oxide nanocarriers FCPZnO = Folic-acid Conjugated *paclitaxel*-filled Zinc-Oxide nanocarriers


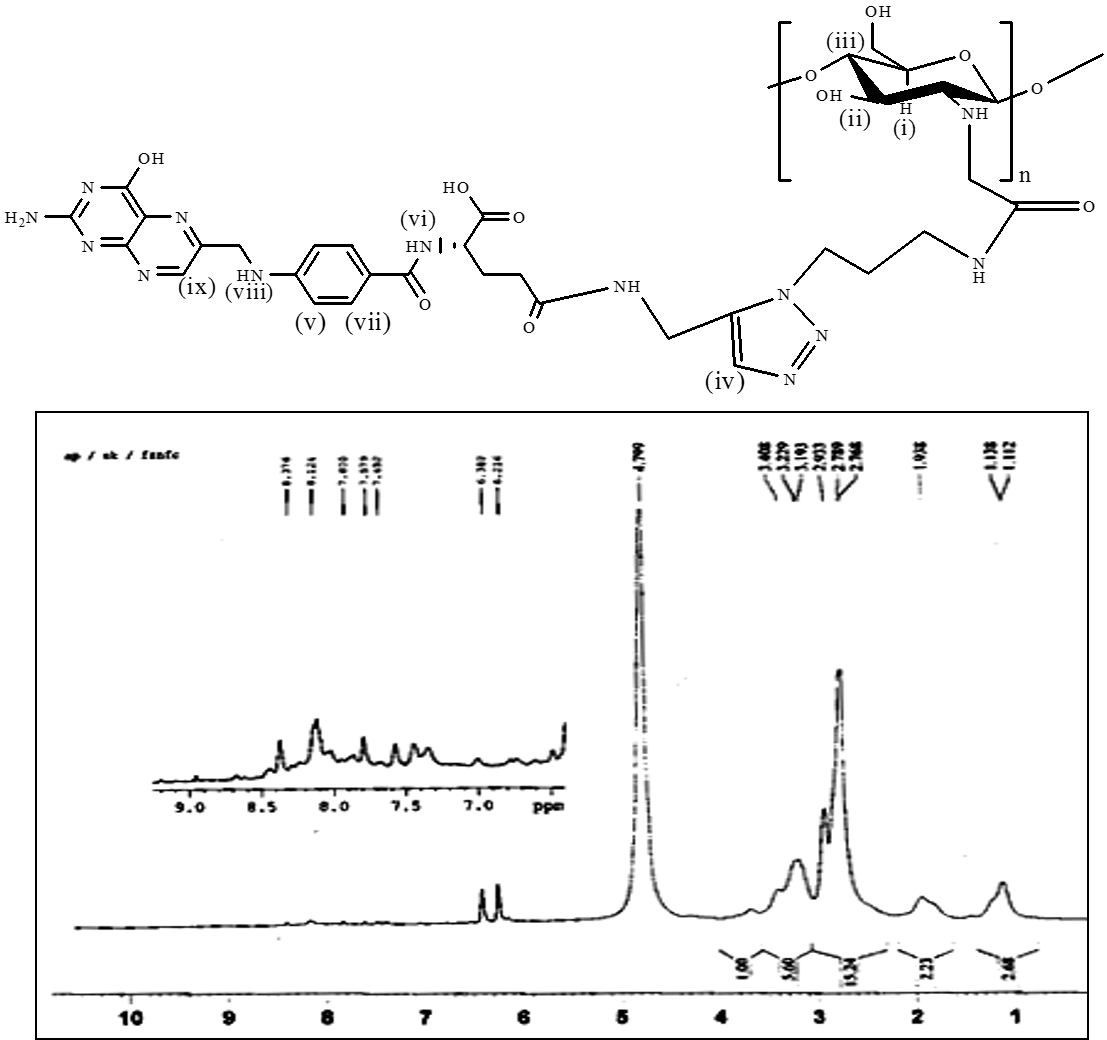


**Fig. S1**.1H NMR spectrum of folic acid conjugated chitosan through click chemistry reaction in absence of hollow ZnO nanospheres.

**Figure S2**

**
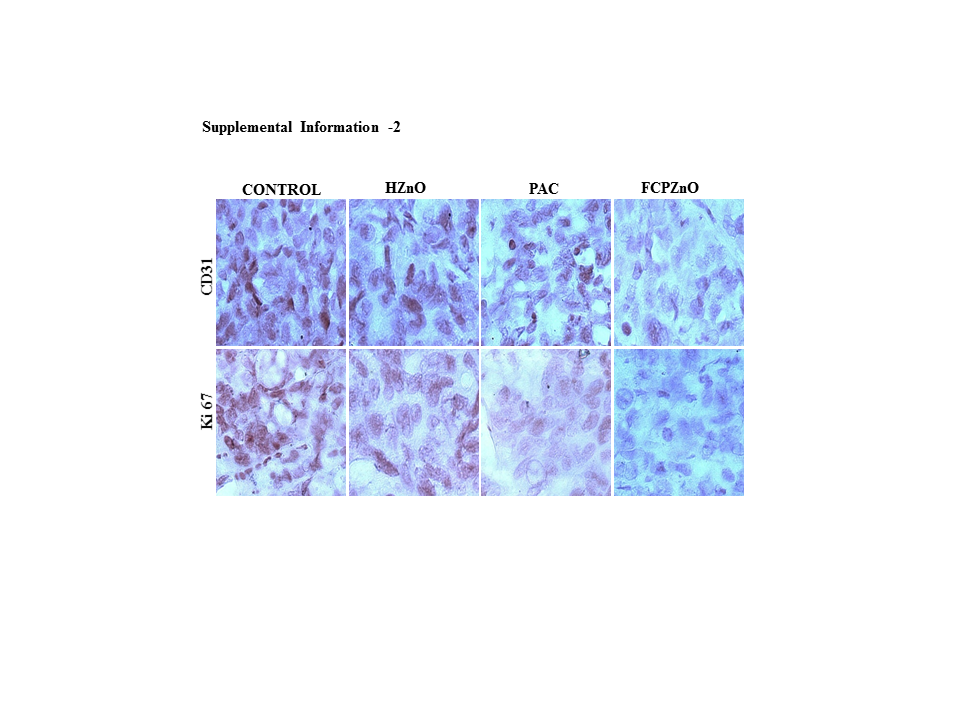
**

**Figure S2:** Tumors from different treatment groups underwent immunohistochemical analysis for expression of CD31 (angiogenesis) and Ki 67 (cell proliferation). Compared with tumors from the control mice, treatment with PAC decreased the number of Ki 67 and CD31 positive cells. These changes were even more prominent when animals were treated with FCPZnO nanospheres. Representative pictures were taken at 20× magnification.

**Figure S3**

**
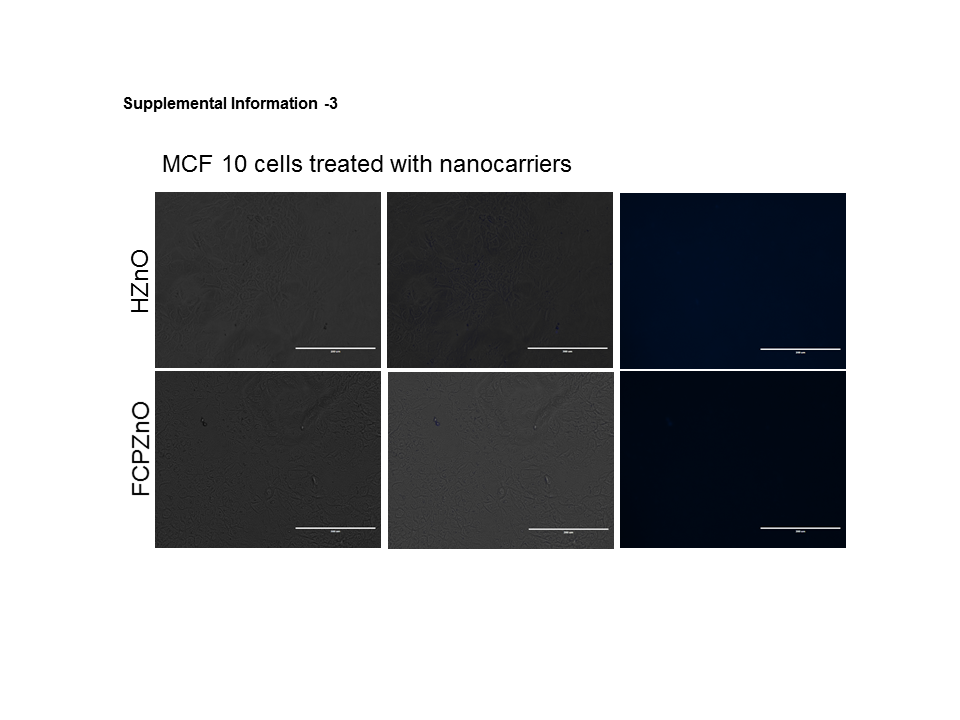
**

**Figure S3:**Cellular uptake studies of MCF10A cells treated with HZnO and FCPZnO nanocarriers for 3 h. Results are representative of three independent experiments. Each Bar represents 20 µm.
